# Clinical validation of the tempus xT next-generation targeted oncology sequencing assay

**DOI:** 10.18632/oncotarget.26797

**Published:** 2019-03-22

**Authors:** Nike Beaubier, Robert Tell, Denise Lau, Jerod R. Parsons, Stephen Bush, Jason Perera, Shelly Sorrells, Timothy Baker, Alan Chang, Jackson Michuda, Catherine Iguartua, Shelley MacNeil, Kaanan Shah, Philip Ellis, Kimberly Yeatts, Brett Mahon, Timothy Taxter, Martin Bontrager, Aly Khan, Robert Huether, Eric Lefkofsky, Kevin P. White

**Affiliations:** ^1^ Tempus Labs Inc., Chicago, IL 60654, USA

**Keywords:** tumor profiling, next-generation sequencing assay validation

## Abstract

We developed and clinically validated a hybrid capture next generation sequencing assay to detect somatic alterations and microsatellite instability in solid tumors and hematologic malignancies. This targeted oncology assay utilizes tumor-normal matched samples for highly accurate somatic alteration calling and whole transcriptome RNA sequencing for unbiased identification of gene fusion events. The assay was validated with a combination of clinical specimens and cell lines, and recorded a sensitivity of 99.1% for single nucleotide variants, 98.1% for indels, 99.9% for gene rearrangements, 98.4% for copy number variations, and 99.9% for microsatellite instability detection. This assay presents a wide array of data for clinical management and clinical trial enrollment while conserving limited tissue.

## INTRODUCTION

Continuous and rapid advances in tumor biology, drug discovery and immunotherapy are accelerating the adoption of precision oncology. There is a growing arsenal of targeted therapeutics that disrupt oncogenes and modulate dysregulated molecular pathways [1]. Additionally, a growing subclass of molecularly targeted immunotherapeutics has developed to either stimulate or reduce the inhibition of cytotoxic T-cells [2–4]. Adoptive T-cell engineering, including chimeric antigen receptor T-cells (CAR-T), is being used to precisely target cancer cells that express specific antigens [5, 6]. Oncolytic viruses are also being engineered to target molecular states of tumor cells [7]. This rapid pace of development has led to a large menu of genomic and transcriptomic alterations that are potentially clinically-relevant for each individual patient. Therefore, well-designed genomic and transcriptomic sequencing panels are necessary for clinical testing [1].

We previously presented the Tempus xO assay, a next generation sequencing (NGS)-based oncology assay that interrogates 1,711 cancer-related genes in matched tumor and normal tissue with whole transcriptome RNA sequencing (RNA-seq) for gene rearrangement detection [8]. We now present the Tempus xT assay, a more focused targeted oncology panel using hybrid capture NGS to interrogate a refined list of 595 genes (Table 1), including solid tumor and hematologic malignancy targets selected through extensive review of recent literature and oncogenic pathway analysis (see Methods). The assay also includes a combination of whole transcriptome RNA-Seq and targeted DNA tiling probes (Table 2) for comprehensive gene rearrangement detection, as well as microsatellite instability (MSI) testing. In addition to the clinical testing function of the assay, the DNA- and RNA-seq assay components support a combination of research and clinical tools for the evaluation of tumor immunity status, including HLA typing, neoantigen prediction, DNA repair gene analysis, MSI status, tumor mutational burden, and immune cell typing and expression.

**Table 1:** xT gene list

ABCB1	ABCC3	ABL1	ABL2	ACTA2	ACVR1B	AJUBA	AKT1	AKT2	AKT3	ALK
AMER1	APC	APOB	AR	ARAF	ARHGAP26	ARHGAP35	ARID1A	ARID1B	ARID2	ARID5B
ASNS	ASXL1	ATIC	ATM	ATP7B	ATR	ATRX	AURKA	AURKB	AXIN1	AXIN2
AXL	B2M	BAP1	BARD1	BCL2	BCL2L1	BCL2L11	BCL6	BCL7A	BCL10	BCL11B
BCLAF1	BCOR	BCORL1	BCR	BIRC3	BLM	BMPR1A	BRAF	BRCA1	BRCA2	BRD4
BRIP1	BTG1	BTK	BUB1B	C3orf70	C8orf34	C10orf54	C11orf30	C11orf65	CALR	CARD11
CASP8	CASR	CBFB	CBL	CBLB	CBLC	CBR3	CCDC6	CCND1	CCND2	CCND3
CCNE1	CD19	CD22	CD40	CD70	CD79A	CD79B	CD274	CDC73	CDH1	CDK4
CDK6	CDK8	CDK12	CDKN1A	CDKN1B	CDKN1C	CDKN2A	CDKN2B	CDKN2C	CEBPA	CEP57
CFTR	CHD2	CHD4	CHEK1	CHEK2	CIC	CIITA	CKS1B	CREBBP	CRKL	CRLF2
CSF1R	CSF3R	CTC1	CTCF	CTLA4	CTNNA1	CTNNB1	CTRC	CUX1	CXCR4	CYLD
CYP1B1	CYP2D6	CYP3A5	DAXX	DDB2	DDR2	DDX3X	DICER1	DIRC2	DIS3	DIS3L2
DKC1	DNM2	DNMT3A	DOT1L	DPYD	DYNC2H1	EBF1	ECT2L	EGF	EGFR	EGLN1
ELF3	ENG	EP300	EPCAM	EPHA2	EPHA7	EPHB1	EPHB2	EPOR	ERBB2	ERBB3
ERBB4	ERCC1	ERCC2	ERCC3	ERCC4	ERCC5	ERCC6	ERG	ERRFI1	ESR1	ETS1
ETS2	ETV1	ETV4	ETV5	ETV6	EWSR1	EZH2	FAM46C	FAM175A	FANCA	FANCB
FANCC	FANCD2	FANCE	FANCF	FANCG	FANCI	FANCL	FANCM	FAS	FAT1	FBXO11
FBXW7	FCGR2A	FCGR3A	FDPS	FGF1	FGF2	FGF3	FGF4	FGF5	FGF6	FGF7
FGF8	FGF9	FGF10	FGF14	FGF23	FGFR1	FGFR2	FGFR3	FGFR4	FH	FHIT
FLCN	FLG	FLT1	FLT3	FLT4	FNTB	FOXA1	FOXL2	FOXO1	FOXO3	FOXP1
FOXQ1	FRS2	FUBP1	G6PD	GALNT12	GATA1	GATA2	GATA3	GATA4	GATA6	GEN1
GLI1	GNA11	GNA13	GNAQ	GNAS	GPC3	GPS2	GREM1	GRIN2A	GRM3	GSTP1
H3F3A	H19	HAS3	HAVCR2	HDAC1	HDAC2	HDAC4	HGF	HIF1A	HIST1H1E	HIST1H3B
HIST1H4E	HLA-A	HLA-B	HLA-C	HLA-DMA	HLA-DMB	HLA-DOA	HLA-DOB	HLA-DPA1	HLA-DPB1	HLA-DPB2
HLA-DQA1	HLA-DQA2	HLA-DQB1	HLA-DQB2	HLA-DRA	HLA-DRB1	HLA-DRB5	HLA-DRB6	HLA-E	HLA-F	HLA-G
HNF1A	HNF1B	HOXB13	HRAS	HSP90AA1	HSPH1	IDH1	IDH2	IDO1	IFIT1	IFIT2
IFIT3	IFNAR1	IFNAR2	IFNGR1	IFNGR2	IFNL3	IKBKE	IKZF1	IL2RA	IL6R	IL7R
IL10RA	IL15	ING1	INPP4B	IRF1	IRF2	IRF4	IRS2	ITPKB	JAK1	JAK2
JAK3	JUN	KAT6A	KDM5A	KDM5C	KDM6A	KDR	KEAP1	KEL	KIF1B	KIT
KLHL6	KLLN	KMT2A	KMT2B	KMT2C	KMT2D	KRAS	LAG3	LDLR	LEF1	LMNA
LMO1	LRP1B	LYN	LZTR1	MAD2L2	MAF	MAFB	MALT1	MAP2K1	MAP2K2	MAP2K4
MAP3K1	MAP3K7	MAPK1	MAX	MC1R	MCL1	MDM2	MDM4	MED12	MEF2B	MEN1
MET	MGMT	MIB1	MITF	MKI67	MLH1	MLH3	MLLT3	MPL	MRE11A	MS4A1
MSH2	MSH3	MSH6	MTAP	MTHFR	MTOR	MTRR	MUTYH	MYB	MYC	MYCL
MYCN	MYD88	MYH11	NBN	NCOR1	NCOR2	NF1	NF2	NFE2L2	NFKBIA	NHP2
NKX2-1	NOP10	NOTCH1	NOTCH2	NOTCH3	NPM1	NQO1	NRAS	NRG1	NSD1	NT5C2
NTHL1	NTRK1	NTRK2	NTRK3	NUDT15	NUP98	P2RY8	PAK1	PALB2	PALLD	PARK2
PAX3	PAX5	PAX7	PAX8	PBRM1	PCBP1	PDCD1	PDCD1LG2	PDGFRA	PDGFRB	PDK1
PDPK1	PHF6	PHOX2B	PIAS4	PIK3C2B	PIK3CA	PIK3CB	PIK3CD	PIK3CG	PIK3R1	PIK3R2
PIM1	PLCG2	PML	PMS1	PMS2	POLD1	POLE	POLH	POT1	POU2F2	PPP1R15A
PPP2R1A	PPP2R2A	PPP6C	PRCC	PRDM1	PREX2	PRKAR1A	PRSS1	PRSS2	PTCH1	PTCH2
PTEN	PTPN11	PTPN13	PTPN22	PTPRD	QKI	RAC1	RAD21	RAD50	RAD51	RAD51B
RAD51C	RAD51D	RAD54L	RAF1	RANBP2	RARA	RASA1	RB1	RBM10	RECQL4	RET
RHOA	RICTOR	RINT1	RIT1	RNF43	RNF139	ROS1	RPL5	RPS6KB1	RPS15	RPTOR
RSF1	RUNX1	RUNX1T1	RXRA	SCG5	SDHA	SDHAF2	SDHB	SDHC	SDHD	SEC23B
SEMA3C	SETBP1	SETD2	SF3B1	SGK1	SH2B3	SLC26A3	SLC47A2	SLIT2	SLX4	SMAD2
SMAD3	SMAD4	SMARCA1	SMARCA4	SMARCB1	SMARCE1	SMC1A	SMC3	SMO	SOCS1	SOD2
SOX2	SOX9	SOX10	SPEN	SPINK1	SPOP	SPRED1	SRC	SRSF2	STAG2	STAT3
STAT4	STAT5A	STAT5B	STAT6	STK11	SUFU	SUZ12	SYK	TAF1	TANC1	TAP1
TAP2	TBC1D12	TBL1XR1	TBX3	TCF3	TCF7L2	TCL1A	TERT	TET2	TGFBR2	TIGIT
TMEM127	TMEM173	TMPRSS2	TNF	TNFAIP3	TNFRSF9	TNFRSF14	TNFRSF17	TOP1	TOP2A	TP53
TP63	TPM1	TPMT	TRAF3	TSC1	TSC2	TSHR	TUSC3	TYMS	U2AF1	UBE2T
UGT1A1	UGT1A9	UMPS	VEGFA	VHL	WEE1	WHSC1	WRN	WT1	XPA	XPC
XPO1	XRCC1	XRCC2	XRCC3	YEATS4	ZFHX3	ZNF217	ZNF471	ZNF620	ZNF750	ZNRF3
ZRSR2										

**Table 2:** DNA gene rearrangement regions

Gene	xT DNA-seq detected regions
ABL1	5′UTR, introns 1, 2
ALK	introns 18, 19, 20
BCR	promoter, UTR, introns 1 through 22
BRAF	introns 8, 9, 10
EGFR	introns 23, 24, 25, 26, 27
ETV6	introns 4, 5
EWSRl	promoter, UTR, introns 1 through 16
FGFR2	5′UTR, introns 1 through 17
FGFR3	Full gene
MYB	Full gene
MYC	Full gene
NRGl	introns 3, 5
NTRKl	introns 9, 10, 11, 12, 13, 14, 15, 16
NTRK3	introns 13, 14
PAX8	Full gene
PDGFRA	intron 11
PML	introns 3, 4, 5, 6, 7, 8
RARA	intron 2
RET	introns 6, 7, 8, 9, 10, 11, 12
ROSI	introns 31, 32, 33, 34, 35, 36, 37, 38, 39
TMPRSS2	UTR, introns 2, 3, 4, 5

## RESULTS

We have instituted performance benchmarks to support the clinical use of the xT assay and have assessed analytical sensitivity, specificity, accuracy and precision across the test's reportable range.

### Single nucleotide variant and indel sensitivity, specificity, and limit of detection

In order to determine assay sensitivity for single nucleotide variants (SNVs) in solid tumors, a panel of formalin-fixed, paraffin-embedded (FFPE) clinical tumor samples were sequenced and compared against previously reported results from the Tempus xO assay [8]. There were 487 unique SNVs previously detected in the tumor samples, with variant allele fractions (VAFs) from 5% to 100% (median 25.9%). All but four variants were detected in both assays, resulting in a SNV sensitivity of 99.1% (354/357). To categorize sensitivity at low VAFs, reference standards containing variants between 1-30% VAF were used (Horizon Diagnostics, Columbus, GA). This comparison showed a sensitivity of 96.9% (126/130).

**Figure 1:**
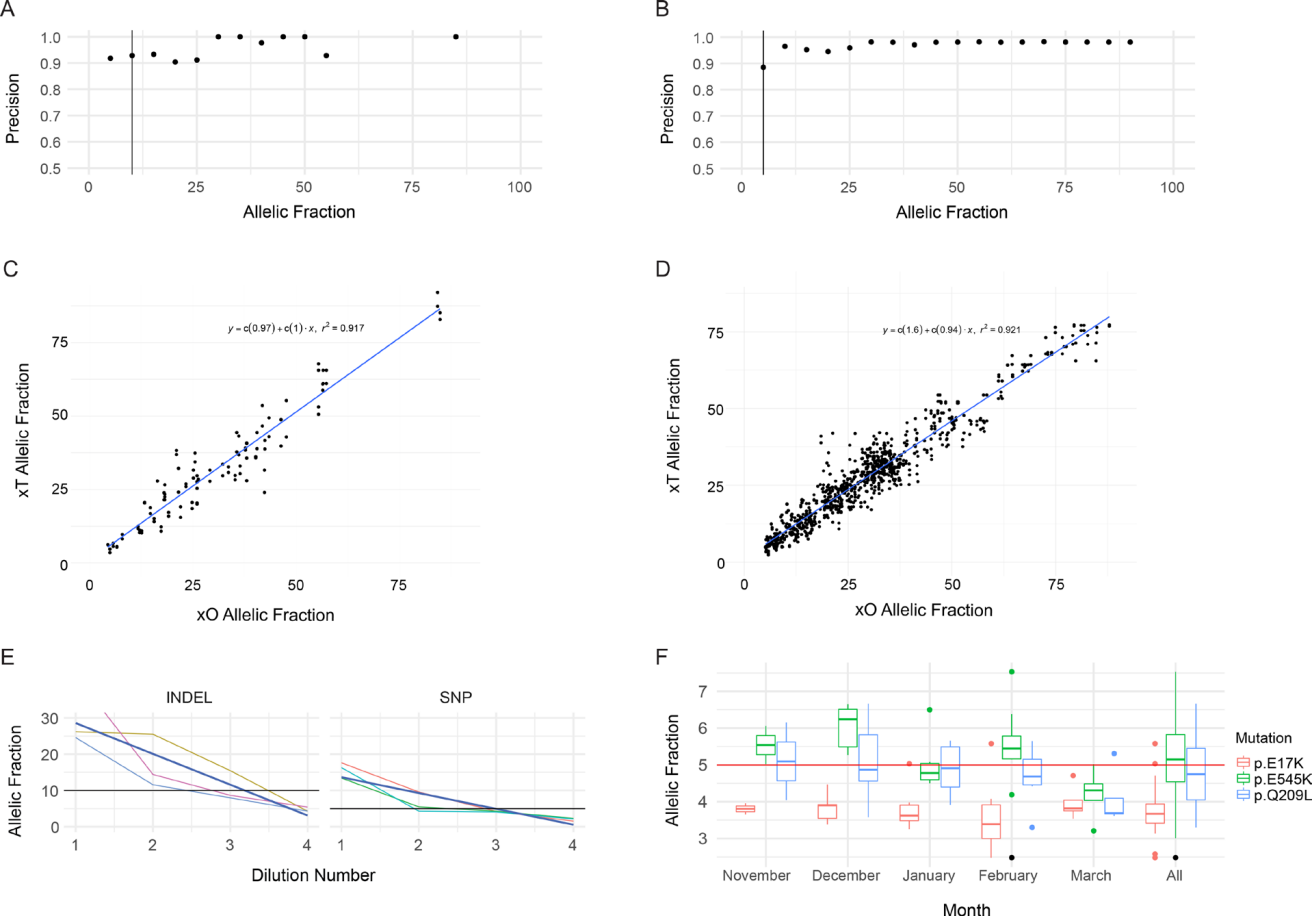
Performance of SNV and Indel Detection by the Tempus xT assay (**A**) Precision of indel detection by VAF. Precision was calculated for each bin of variants with allele fractions rounded to the nearest 5 percent. The vertical black line corresponds to the LOD. (**B**) Precision of SNV detection by VAF, as in A. (**C**) Correlation of xT assay indel VAFs to xO assay indel VAFs. (**D**) Correlation of xT assay SNV VAFs to xO assay SNV VAFs. (**E**) VAFs of indels and SNVs detected on chromosome 17 in four samples with serial 1:1 dilutions of the xT assay. Dark blue lines indicate best fit of a linear model. (**F**) Positive control detection. Boxplots of the VAF of three SNVs in a positive control sample run on every xT assay over a period of 5 months. The single point marked in black is an *AKT1* p.E17K variant which failed filtering criteria.

Specificity was calculated as the number of bases identified as negative for variation by both the xT assay and orthogonal methodology, divided by the total number of bases called negative by the assay. A total of two false positives were observed resulting in a specificity of >99.9%, with a positive predictive value (PPV) of 99.5% (347/349). The overall precision of the assay was calculated as 97.2%, with a slight dependency on base fraction at the lower limit of detection (LOD) (Figure 1A, 1B). Additionally, the correlation of VAF measurements between the xT and xO assays was determined by measuring the correlation coefficient (Figure 1C, 1D). The xT assay shows high concordance in base fractions (r^2^ = 0.971 for indels, r^2^ = 0.921 for SNVs) with the xO assay at all ranges of performance.

Serial dilution of tumor samples with matched normal samples was performed to generate variants with base fractions between 25% and 1% VAF. Three variants were detected using dilutions down to 1.4% VAF (1:16 dilution of a 20% variant in the tumor). One mutation was detected down to an allele fraction of 2.1% (1:8 dilution) but not at the 1:16 dilution (Figure 1E). A conservative LOD of 5% was therefore set for SNVs, although we observed consistent detection below that threshold (Figure 1F).

Indels were consistently detected down to 4% VAF (Figure 1E). A conservative LOD of 10% was therefore set for the indels. Fifty indels were called within the xT LOD for 48 samples in the set. Of these 50 indels, 48 were called by xT, and one variant (*NOTCH3* p.1317fs) was excluded from analysis due to insufficient coverage on the xT panel. Thus, the final sensitivity calculation was 98.0% (48/49). PPV was calculated using the 49 indels called by the xT assay and their comparison against all xO data. All 49 indels called were concordant with the xO assay. We, therefore, observed a >99.9% PPV for indel detection.

### Gene rearrangement and fusion validation

**Figure 2:**
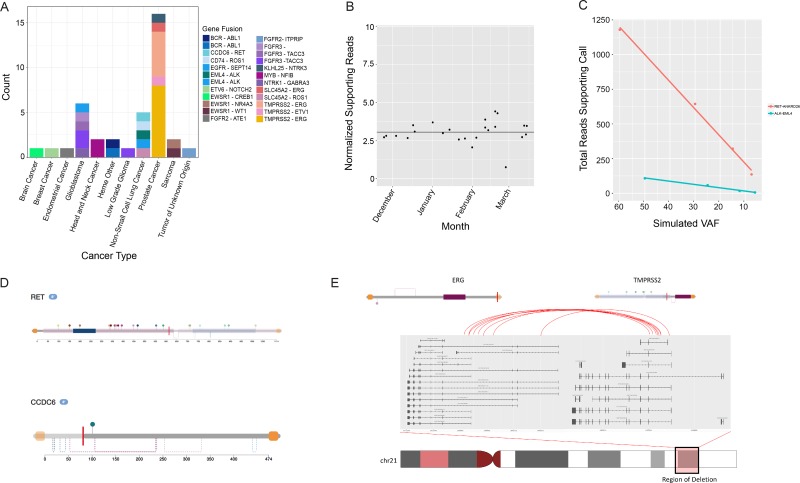
Analysis of rearrangement detection performance by the Tempus pipeline and retrospective analysis of recurrent fusions (**A**) Fusions detected by cancer type. (**B**) Positive control fusion samples (*ROS1-SLC34A2*) processed by the xT assay over the course of 4 months. The fusion was expected at 5% VAF in the control and was consistently detected across flow cells and instruments. (**C**) Limit of detection analysis for two serially diluted fusions (*ALK-EML4* and *RET-ANKRD26*). Both fusions were detected down to 3–5% simulated VAF by the Tempus xT Assay. (**D**) Functional characterization of fusions detected by the xT assay. Domains, regions, and sites are highlighted for orientation along the amino acid sequence for each protein involved in the rearrangement event. (**E**) Analysis of the recurrent *TMPRSS2-ERG* fusion found in 13 prostate cancers detected by the xT assay. The xT assay consistently localized breakpoints to the expected functional domains resulting in the *TMPRSS2* promoter and replacing the first several exons of *ERG* by a chromosomal deletion. This results in the functional domains of *ERG* being largely intact, but under the control of the *TMPRSS2* promoter.

The assay is designed to assess 21 gene rearrangement targets by DNA-seq (Table 2), in addition to comprehensive fusion detection by RNA-seq as previously reported [8]. The reportable range for gene rearrangements by DNA-seq is limited to fusions occurring in the specific regions listed in Table 2. Twenty-seven validation samples (including 23 patient samples and 4 reference standards) with known gene rearrangements were sequenced. Results were compared with the previously validated RNA-seq fusion detection assay [8], and the reference standard results were compared with the manufacturer-provided data sheets. The assay successfully detected 28 of the 29 gene rearrangements within the 27 samples. The DNA-seq translocation detection sensitivity was 96.5% (28/29), with an overall sensitivity of translocation detection, including RNA-seq, of 99.9% (29/29). The overall distribution of reported gene rearrangements by cancer type for the patient cohort sequenced at Tempus Labs is shown in Figure 2A.

Specificity was analyzed with a subset of 13 samples that were assessed for the absence of additional xT reportable fusions. This was calculated as the number of samples (*n* = 13) multiplied by the number of assayed genomic sites (*n* = 21) for 273 positions. The xT assay called zero false positive translocations, resulting in a >99.9% specificity. Furthermore, a positive control fusion monitored by Tempus (*ROS1-SLC34A2*) in the reference standard (HD753, Horizon Diagnostics) was detected 20 out of 20 times (Figure 2B).

To assess the LOD, we used a serial dilution of two known positive control samples (containing *ALK-EML4* and *ANKRD26-RET*, respectively). Samples were diluted from 50-60% VAF in the primary sample to 3-4% VAF (Figure 2C). The gene rearrangements were detected in all experiments, but in the case of the two lowest titrations for *ALK-EML4*, the number of supporting reads fell below the normal reporting threshold for the assay. A conservative LOD was set at 10% based on the *ALK-EML4* detection data. Additionally, we functionally characterized clinically relevant fusions detected via the xT assay, such as *RET-CCDC6* and *TMPRSS2-ERG* fusions (Figure 2D, 2E).

### Copy number alterations

Copy number variation (CNV) is particularly difficult to detect in targeted panels. Paired-end mapping strategies typically fail because the majority of CNV breakpoints occur in non-targeted regions [20]. The depth of coverage is the primary metric used to determine copy number, but variable probe affinities, probe balance, and hybridization produce significant coverage variability [21]. This can be corrected by comparing the tumor sample with its matched normal sample and/or a pool of unrelated normal samples. The xT panel design significantly reduces the number of heterozygous SNVs required to make an integrated segmentation call. Accordingly, models used for fitting corrected coverage ratios to potential ploidy and corresponding copy number are selected based not only on goodness-of-fit but also on the resulting genome-wide ploidy state [22]. CNV specificity was assessed as the total number of genes assayed (*n* = 67 patients, 39 genes each) and the number of false positive detections (*n* = 3). Specificity was calculated as called negatives/true negatives, resulting in a final specificity of 99.8%. PPV was calculated as the total number of amplification calls made by the xT copy number analysis pipeline (*n* = 70) versus the number of copy number calls that were correctly identified as amplified (*n* = 67). This latter analysis was performed using CNVs that were clearly amplified in xO (>9 copies), or those identified as amplified in xT, but moderately amplified (>5 copies) in xO. This resulted in a final PPV of 95.7%. To assess LOD, three samples with CNVs in *ERBB2, CDK12,* or *EGFR* were diluted between 50% and 5% tumor purity. In all cases, amplified regions were detected and identified as amplified down to at least 12.5% tumor purity. To allow for the detection of less-heavily amplified genetic regions, a lower LOD was set at 30% tumor purity.

**Figure 3:**
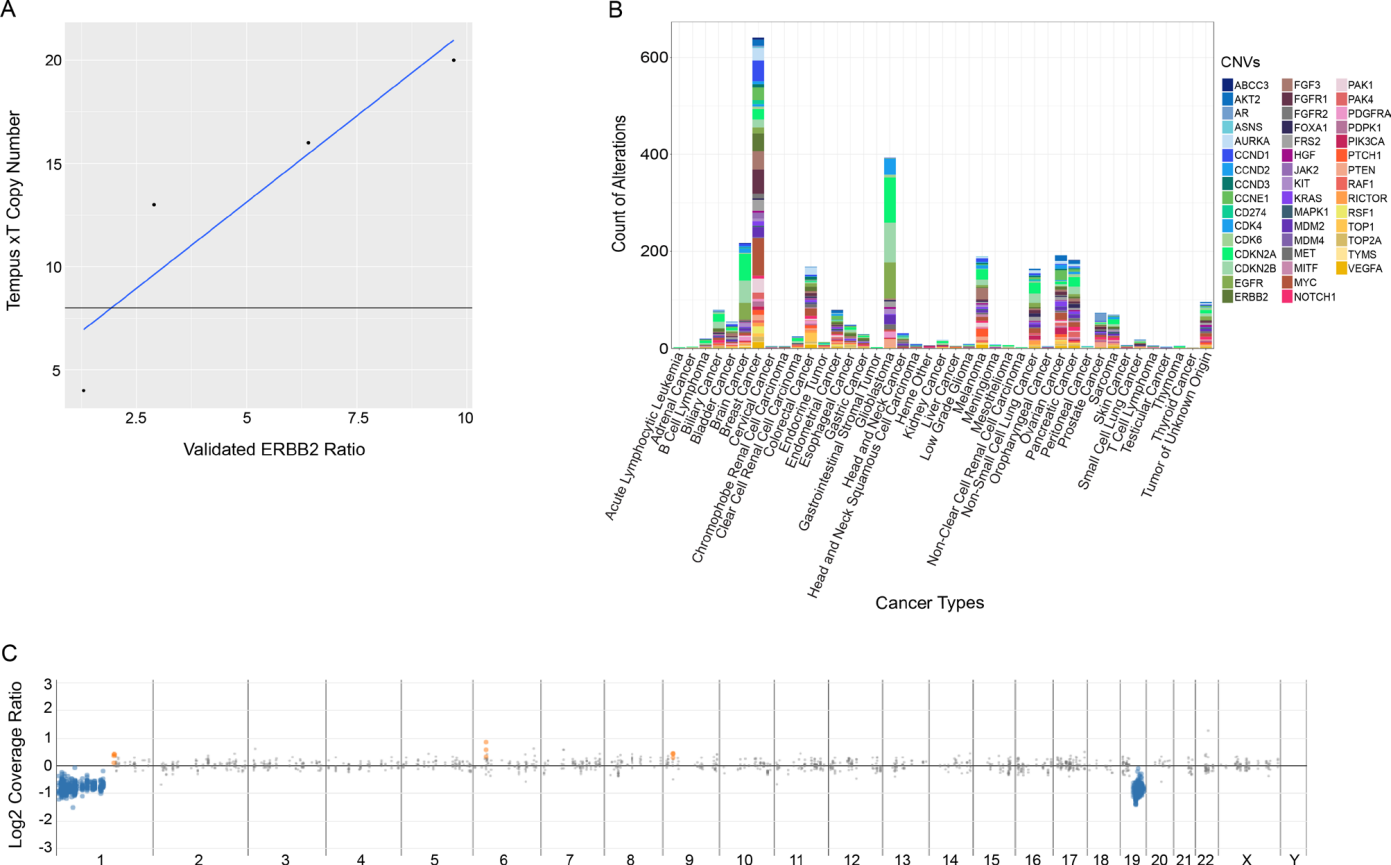
Analysis of performance of the xT assay in the detection of copy number variations (**A**) Detection comparison of Tempus copy number calls against validated *ERBB2* control ratios. (**B**) Plot of reported CNVs by cancer type for the xT patient cohort sequenced at Tempus. (**C**) A representative 1p-19q co-deletion detected commonly in oligodendrogliomas. The blue dots represent genomic regions showing one copy loss while the grey points represent neutral segments.

Additionally, the National Institutes of Standards and Technology's (NIST) RM 2373 (Genomic DNA Standards for HER2 Measurements) was evaluated for CNVs in *ERBB2* (HER2). The copy number results for *ERBB2* generated by the xT assay were linear (r^2^=0.97) with respect to the validated copy ratios across the five reference samples (Figure 3A). Furthermore, the amplification call crosses the regression line at an *ERBB2* ratio of 2.5. In calling *ERBB2* positivity in breast cancers using FISH, an *ERBB2* ratio of 2.2 was used to call positivity, closely correlating with the assay threshold for amplification calling. The distribution of CNVs by cancer type for the patient cohort sequenced at Tempus Labs is shown in Figure 3B.

Finally, large scale genomic alterations were assessed using the xT assay (Figure 3C). Due to the long-range genomic tiling of the xT assay, it was hypothesized that large scale genomic instability might be detectable via chromosome level copy number visualization. For example, in oligodendrogliomas, the xT assay consistently detected the 1p-19q co-deletion, an important diagnostic and prognostic marker. These complex structural alterations were successfully detected with the xT assay although they are not part of the common class of focal alterations reported in the literature from NGS-based tumor profiling methodologies.

### Microsatellite instability detection

MSI results from defects in DNA mismatch repair. To validate MSI status determination and evaluate the accuracy of the test, we analyzed samples from 34 patients (14 microsatellite instability-high [MSI-H], 20 microsatellite stable [MSS]). For three MSI-H patients, an additional four samples were generated for LOD testing with a two-fold serial dilution. For two additional MSI-H patients, three replicates were sequenced on the same plate for intra-assay precision and three replicates were sequenced on separate plates for inter-assay precision. The accuracy, precision, and LOD were assayed for tumor-normal paired and tumor-only samples.

The xT MSI assay was validated against an MSI PCR assay using the five marker Bethesda panel (Arup Laboratories, Salt Lake City, UT), a four-protein MMR immunohistochemistry (IHC) panel (Tempus Labs, Chicago, IL), or both. Samples found to be MSI-H by the MSI PCR, or missing expression in at least one protein by the MMR IHC were collectively considered MSI-H. Similarly, samples found to be MSS by the MSI PCR, or with normal MMR protein expression by the MMR IHC were considered MSS. One sample had contradictory MSI PCR and MMR IHC results and was removed from the study. In both paired and unpaired modes, the 14 MSI-H samples were correctly classified as MSI-H and the 20 MSS samples were correctly classified as MSS, thus, the sensitivity of MSI status determination was >99%, the specificity was >99%, and the PPV was >99%.

To establish the LOD, three MSI-H patient DNA samples were serially diluted to determine the minimum tumor fraction necessary to reliably detect MSI-H status. MSI-H status was consistently detected down to approximately 20% tumor in both the paired and unpaired modes. The LOD was conservatively set at 30% tumor.

### Immuno-oncology profiling

Immunotherapy has become a key tool for treating a wide range of cancers. A current challenge in the field is the proper identification of patients most likely to benefit from this powerful but expensive therapy that can also have severe side effects [25–28]. In the course of clinical care, TMB, Human Leukocyte Antigen (HLA) type, neoantigen load, and MSI status were calculated in order to evaluate the benefits of immunotherapy. HLA genes are involved in the presentation of self- and foreign peptides to T cells. Specific HLA alleles are associated with serious pharmacological counter-indications, eligibility for clinical trials, and increased probability of response or non-response to checkpoint therapy. HLA typing is also a prerequisite for neoantigen prediction. We assessed the accuracy and sensitivity of *in silico* HLA typing on a set of 72 known reference samples obtained directly from the International Histocompatibility Working Group in Seattle, WA (www.ihwg.org). For reference samples sequenced on the xT panel, class I HLA typing was 99.8% accurate at the two-digit resolution and 96.6% accurate at the four-digit resolution (Supplementary Table 1). Sequencing was also sensitive for alleles associated with pharmacological counter-indications and alleles used for inclusion and exclusion criteria in immunotherapy clinical trials (Supplementary Tables 2–3).

**Figure 4:**
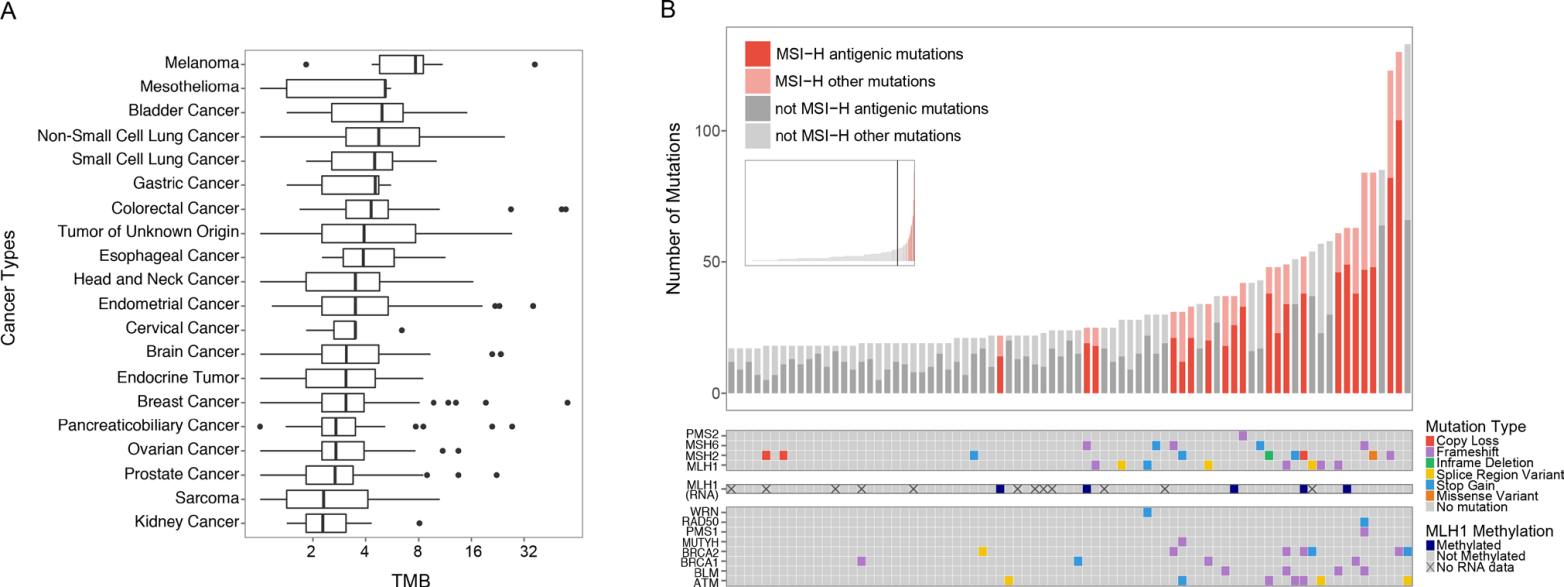
Survey of immunotherapy markers across diverse cancer types (**A**) The distribution of TMB for each cancer type plotted on a log2 scale and ordered by the median TMB. Outliers (data points beyond 1.5x interquartile range) are shown as individual points. (**B**) Analysis of samples in the top 10th percentile of TMB. The inset shows the distribution of TMB across all samples included in the study, with the vertical bar marking the 90th percentile of TMB. The bar chart shows the proportion of predicted antigenic mutations of the non-synonymous mutations detected. MSI-H samples are highlighted in red. The color-coded matrices show the MMR gene mutations detected by mutation type (top), predicted MLH1 methylation status (middle), and DNA repair gene mutations detected (bottom).

TMB and MSI status was assessed in 806 clinical samples spanning more than 28 different cancer types (Figure 4). While predictive power varied with cancer type, the xT assay TMB scoring recapitulated disease-specific TMB estimates previously reported in the literature [29] (Figure 4A). One of the most well characterized mechanistic drivers of high TMB is MSI-H status. The xT MSI assay showed that MSI-H tumors accounted for 26.6% of tumors in the top decile of TMB, with MSI-H status significantly associated with high TMB (*p* = 8.72e–26, hypergeometric test). In MSI-H cases, alterations were frequently found in genes encoding DNA mismatch repair (MMR) proteins (MLH1, PMS2, MSH2, and MSH6). In a number of cases where no genetic alterations were found, we were able to detect MLH1 silencing based on reduced RNA expression. The assay also detected an enrichment of alterations in known DNA repair genes, including *WRN, RAD50, PMS1, MUTYH, BRCA1, BRCA2, BLM,* and *ATM* (Figure 4B).

### Pan-cancer molecular profiling

**Figure 5:**
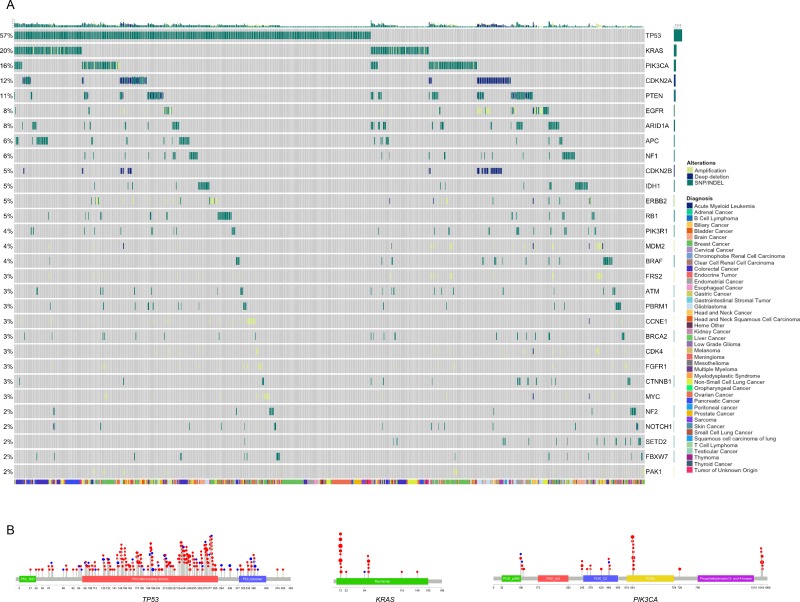
Mutational landscape across all cancer types (**A**) Plot of a subset of patients within the Tempus xT cohort containing at least one biologically relevant alteration, stratified by alteration prevalence. Patients were clustered by the mutational profile similarity of genes (y-axis) with at least 5 actionable alterations detected within the cohort. Patient cancer type is displayed by the colored bar below the matrix. (**B**) Lollipop plot of *TP53, KRAS*, and *PIK3CA* alterations detected by the xT assay across all cancer types.

We next analyzed the landscape of genomic alterations in 1074 clinical samples assayed with the xT panel. A subset of samples was optimized to appropriately represent the percentages of cancer types seen in clinical care sites serviced by Tempus Labs. These data were then compared against large-scale genomic profiling efforts [23, 24] to assess the clinical validity of the assay. Within the Tempus cohort, 952 samples contained at least one biologically relevant alteration (88.6%), which was defined as an alteration associated with pathogenicity based on literature, databases, or *in silico* reviews. The evidence for biological relevance ranges from alterations addressed by the National Comprehensive Cancer Network (NCCN) guidelines with FDA-approved therapeutic interventions, through on and off tissue clinical research, down to preclinical evidence with or without clinical trial eligibility. Within the 952 patients, the most prevalent alterations across cancer types were the tumor suppressor *TP53* (57%) and the oncogene *KRAS* (20%) (Figure 5A). Along with these highly prevalent alterations known to be present in an array of cancer types, there were many canonical oncogenic pathway mutations detected, including gain of function mutations in oncogenes (*EGFR* 11%, *PIK3CA* 16%) and loss of function mutations in tumor suppressor genes (*PTEN* 11%, *ARID1A* 8%, *APC* 6%) [37, 38, 41]. Furthermore, the localization of variants in recurrently mutated genes showed a strong correlation across cancer types (Figure 5B), indicating consistent functional mechanisms for oncogenicity as expected from previous pan-cancer studies [24].

## DISCUSSION

Molecularly targeted therapies, including immunotherapy, are providing better treatment options for cancer patients. To fully utilize these advances, patients must undergo broad molecular tumor profiling for optimal, personalized treatment selection [1]. According to NCCN guidelines, therapy targeted towards specific molecular alterations is already a standard of care in several tumor types, including melanoma, colorectal cancer, and non-small cell lung cancer. These few, well-known mutations could be detected with individual assays or small NGS panels. However, for the largest number of patients to benefit from personalized oncology, molecular alterations that can be targeted with off-label drug indications, combination therapy, or tissue agnostic immunotherapy should be assessed [30–32]. Large-panel NGS assays also cast a wider net for clinical trial enrollment [33, 34]. Recent studies indicate that clinical care is guided by NGS assay results for 30-40% of patients who receive such testing [35–38].

We have developed a hybrid capture NGS-based assay to accurately detect clinically relevant alterations across 595 genes that are carefully curated to address solid tumors and hematologic malignancies, plus perform genome-wide unbiased fusion detection. The assay inputs include FFPE tissue, blood or bone marrow tumor specimens, and blood or saliva for germline testing. This assay is unique in its use of matched tumor and normal DNA plus whole transcriptome RNA-seq to provide a comprehensive overview of somatic genomic alterations, including MSI status for targeted cancer therapy, immuno-oncology, and clinical trial enrollment. The test was validated by multiple testing modalities, including a comparison of patient samples to reference assays and pooled cell-line models spanning key determinants of detection accuracy for somatic alterations such as VAF, indel length, the degree of stromal admixture, and amplitude of CNV.

Large NGS panels optimize targeted therapy options because they reveal a wide range of genomic alterations and can be used when working with small FFPE tissue samples. Additionally, broad-based NGS genomic profiling enables patients with rare genomic alterations to be identified for clinical trials. The Tempus xT assay provides the opportunity to deep screen known actionable gene variants and a broad set of biologically relevant cancer-related genes on a clinically validated platform with a relatively rapid turnaround time.

## MATERIALS AND METHODS

### xT assay gene selection

Genes were selected for the xT assay based on recommendations from major professional oncology-related societies, including the National Comprehensive Cancer Network (NCCN), the Association for Molecular Pathology (AMP), the College of American Pathologists (CAP), and the American College of Medical Genetics and Genomics (ACMG). An extensive literature review was conducted to include the addition of genes from known oncogenic pathways, particularly those covered in The Cancer Genome Atlas (TCGA) analysis of oncogenic signaling pathways [41]. In this publication, 89% of the 9,125 tumors analyzed contained at least one driver alteration in one of the 10 canonical pathways. Thus, these genes alone account for a substantial number of driver mutations across cancer types. Next, genes from DNA repair, chromatin remodeling, splicing, ubiquitination, and metabolic pathways were included in the assay. Genes associated with treatment resistance and cancer predisposition, including all cancer-associated genes listed as incidental findings by the ACMG, were also included. Additionally, the intronic regions of 21 key genes that undergo clinically relevant gene rearrangements were included for robust fusion detection. Finally, the gene list was reviewed for completeness by experts familiar with the literature and current medical practices for all major tumor types.

### Sample processing and nucleic acid extraction

A total of 265 patient samples were processed and used in the validation of this study. Normal blood samples were collected in PAXgene Blood DNA Tubes (Catalog #761115) and saliva samples were collected in Oragene DNA Saliva Kits (Catalog #OG-510). Germline (“normal”) DNA was extracted from either 650μl of saliva or 200 μl of blood. After expert pathologist assessment of overall tumor amount and percent tumor cellularity as a ratio of tumor to normal nuclei met a 20% threshold, solid tumor total nucleic acid was extracted from macrodissected FFPE tissue sections and digested by proteinase K. RNA was purified from the total nucleic acid by DNase-I digestion.

Hematologic malignancy samples were collected in EDTA collection tubes. We examined a set of samples collected at Rush University Medical Center (RUMC) that were also analyzed with a Genoptix Myeloid Molecular Panel (Carlsbad, CA) at RUMC. A subset of samples was also sent to Mayo Medical Laboratories (Rochester, MN) for additional testing with a 33 gene hematologic profiling panel.

### DNA and RNA library construction and sequencing

DNA and RNA sequencing was performed as previously described [8]. Briefly, 100 nanograms (ng) of DNA for each tumor and normal sample was mechanically sheared to an average size of 200 base pairs (bp) using a Covaris ultrasonicator. DNA libraries were prepared using the KAPA Hyper Prep Kit, hybridized to the xT probe set, and amplified with the KAPA HiFi HotStart ReadyMix. One hundred ng of RNA for each tumor sample was heat fragmented in the presence of magnesium to an average size of 200 bp. Library preps were hybridized with the IDT xGEN Exome Research Panel and target recovery was performed using Streptavidin-coated beads, followed by amplification with the KAPA HiFi Library Amplification Kit. The amplified target-captured DNA tumor libraries were sequenced to an average unique on target depth of 500x on an Illumina HiSeq 4000. Samples were further assessed for uniformity with each sample required to have 95% of all targeted bp sequenced to a minimum depth of 300x.

### Detection of somatic variants by the xT assay

Tumor and normal FASTQ files were matched to their appropriate pair. FASTQ files were analyzed using FASTQC for rapid assessment of quality control and aligned with Novoalign (Novocraft, Inc.). The SAM files were converted to BAM, BAM files were sorted, and duplicates were marked. Following alignment and sorting, SNVs were called. To assess copy number, de-duplicated BAM files and a VCF generated from the variant calling pipeline were processed for computation of read depth and variation in heterozygous germline SNVs between the tumor and normal samples (or between the tumor sample and a pool of process matched normal controls for tumor-only cases). Circular binary segmentation [9] was applied and segments were selected with highly differential log2 ratios between the tumor and its comparator. Approximate integer copy number was then assessed from a combination of differential coverage in segmented regions and an estimate of stromal admixture generated by analysis of heterozygous germline SNVs.

### Detection and visualization of gene rearrangements by the xT assay

Following de-multiplexing, tumor FASTQ files were aligned against the human reference genome using BWA for DNA files, or aligned to GRCh38 using STAR for RNA files [10, 39]. Raw RNA read counts were then normalized to correct for GC content and gene length using full quantile normalization and adjusted for sequencing depth via the size factor method. DNA reads were sorted and duplicates were marked with SAMBlaster [40]. Discordant and split reads were further identified and separated. These data were then read into LUMPY [11] for structural variant detection. Structural alterations were grouped by type, recurrence, and presence within the Tempus database and displayed through the Tempus quality control application (TSQC) fusion tool. The TSQC fusion viewer referenced Ensembl to determine the gene and proximal exons surrounding the breakpoint for any possible transcript generated across the breakpoint. It then placed the breakpoint 5′ or 3′ to the subsequent exon in the direction of transcription. For inversions, this orientation was reversed for the inverted gene. After positioning of the breakpoint, the translated amino acid sequences were generated for both genes in the chimeric protein, and a plot was generated containing the remaining functional domains for each as returned from Uniprot [12] (Figure 2D, 2E).

### Variant classification and reporting

Variants were investigated following criteria from known evolutionary models, functional data, clinical data, and literature. Variants were then prioritized and classified based on known gene-disease relationships, hotspot regions within genes, internal and external somatic databases, primary literature, and other features of somatic drivers [13, 14, 15]. Variants were reported based on recommendations from the AMP/ASCO/CAP guidelines [16]. Briefly, pathogenic variants with therapeutic, diagnostic, or prognostic significance were prioritized in the report. Non-actionable pathogenic variants were included as biologically relevant, followed by variants of uncertain significance. Translocations were reported based on features of known gene fusions, relevant breakpoints, and biological relevance. Evidence was curated from outside sources and presented as 1) consensus guidelines 2) clinical research, or 3) case studies, with a link to the supporting literature. Germline alterations were reported as secondary findings in a subset of genes for consenting patients. These include genes recommended by the ACMG [17] and additional genes associated with cancer predisposition or drug resistance.

### Microsatellite instability status

We developed probes for 43 microsatellite regions for the xT assay. The MSI classification algorithm classifies tumors into three categories: microsatellite instability-high (MSI-H), microsatellite stable (MSS), or microsatellite equivocal (MSE). MSI testing for paired tumor-normal patients used reads mapped to the microsatellite loci with at least five bp flanking the microsatellite. The identification of at least 30 mapping reads in both tumor and normal samples were required for the locus to be included in the analysis. At least 20 of the 43 microsatellites on the panel were required to reach the minimum coverage. Each locus was individually tested for instability, as measured by changes in the number of repeats in tumor data compared to normal data, using the Kolmogorov-Smirnov test. If *p* ≤ 0.05, the locus was considered unstable. The proportion of unstable microsatellite loci was fed into a logistic regression classifier trained on samples from the TCGA colorectal and endometrial cohorts, which have clinically determined MSI statuses. For MSI testing in tumor-only mode, the mean and variance for the number of repeats were calculated for each microsatellite locus. A vector containing the mean and variance data was put into a support vector machine classification algorithm. Both algorithms returned the probability of the patient being MSI-H. If there was a >70% probability of MSI-H status, the sample was classified as MSI-H. If there was between a 30-70% probability of MSI-H status, the test results were too ambiguous to interpret and those samples were classified as MSE. If there was a <30% probability of MSI-H status, the sample was considered MSS.

### Tumor mutational burden

TMB was calculated by dividing the number of non-synonymous mutations by the megabase size of the panel (2.4 MB). All non-silent somatic coding mutations, including missense, indel, and stop-loss variants, with coverage >100x and an allelic fraction >5% were counted as non-synonymous mutations. A TMB >9 mutations per million bp of DNA was considered “high”. This threshold was established by hypergeometric testing for the enrichment of tumors with orthogonally defined hypermutation (MSI-H) in the larger Tempus clinical database.

### HLA typing

HLA class I typing was performed using Optitype on DNA sequencing including class I HLA-mapped reads and unmapped reads [18]. Normal samples were used as the default reference for matched tumor-normal samples. Tumor sample-determined HLA type was used when the normal sample did not meet internal HLA coverage thresholds, or there was no matched normal sample.

### Neoantigen prediction

Neoantigen prediction was performed on all non-silent mutations. The binding affinities for all possible 8-11 amino acid (aa) peptides containing the mutation were predicted using MHCflurry [19]. For alleles with insufficient training data to generate an allele-specific MHCflurry model, binding affinities were predicted from the nearest HLA allele as assessed by aa homology. A mutation was determined to be antigenic if any resulting peptide was predicted to bind to any of the patient's HLA alleles with <500 nM affinity.

## SUPPLEMENTARY MATERIALS TABLES


