# Supplementary material for: Clinical validation of the tempus xT next-generation targeted oncology sequencing assay
Source: Oncotarget. 2019 Mar 22;10(24):2384–96. doi: 10.18632/oncotarget.26797 (PMC6481324; doi:10.18632/oncotarget.26797)
Supplement: Supplementary file 1 [file oncotarget-10-2384-s001.pdf]

Clinical validation of the tempus xT next-generation targeted oncology sequencing assay

SUPPLEMENTARY MATERIALS

Supplementary Table 1: Class I HLA typing accuracy

Gene	Two-digit accuracy	Four-digit accuracy	Zygosity accuracy
**HLA-A**	99.29%	94.29%	97.14%
**HLA-B**	100.00%	97.14%	97.14%
**HLA-C**	100.00%	96.43%	97.14%
**All Class I**	99.76%	96.61%	97.14%

Accuracy of *in silico* predicted HLA types determined for 72 reference samples acquired directly from the International Histocompatibility Working Group. Accuracy is calculated as the percentage of correctly identified alleles. Zygosity was calculated as an independent metric measuring whether heterozygosity or homozygosity was correctly predicted.

Supplementary Table 2: Class I HLA alleles associated with clinical trial criteria

Use	Allele	Relevant clinical trials (NCTID)	Present	Detected	False positive
**Inclusion Criteria**	A*01 (family)	NCT02153905	13	13	0
	A*02 (family)	NCT03132922, NCT00194714, NCT00060528, NCT01876212, NCT02869217	27	27	0
	A*02:01	NCT02869217, NCT02408016, NCT03247309, NCT02989064, NCT03132792, NCT03326921, NCT00509288, NCT02858310	14	14	2*
	A*02:06	NCT02869217, NCT02989064	2	2	0
	A*11:01	NCT03190941	7	6**	0
	C*08:02	NCT01174121	5	5	0
**Exclusion Criteria**	A*02:02	NCT03132792	1	1	0
	A*02:05	NCT02153905, NCT02989064	1	1	0
	A*02:07	NCT02153905	1	1	0
	B*15:01	NCT02989064	6	6	0
	B*46:01	NCT02989064	4	4	0
	B*51:03	NCT03132792	1	1	0
	C*04:04	NCT03132792	0	0	0

HLA prediction accuracy on specific alleles that are mentioned as inclusion or exclusion criteria in clinical trials listed on ClinicalTrials.gov. For reference samples known to carry these alleles, we assessed how frequently HLA prediction also identified these alleles as well as the rate of false positive calls.

*Both false positives are rare null alleles, one of which is identical across the exons assayed and the other differs by only 1 nucleotide.

**Illumina in their validation of TruSight HLA v2 and HLAassign both had difficulty detecting the presence of A*11:01 in this sample and were unable to confirm A*11 in the missed call by close inspection of HLA reads or Luminex Assay.

Supplementary Table 3: Class I HLA alleles associated with pharmacological adverse events

Allele	Drug interaction	Present	Detected	False positive
**A*31:01**	Carbamazepine[1]	2	2	0
**B*15:02**	Carbamazepine[1], Oxcarbazepine[2], Phenytoin[3]	1	1	0
**B*57:01**	Abacavir[4], Pazopenib[5]	2	2	0
**B*58:01**	Allopurinol[6]	4	4	0

HLA prediction accuracy on specific alleles that are associated with pharmacological adverse events. For reference samples known to carry these alleles, we assessed how frequently HLA prediction identified these alleles as well as the rate of false positive calls.
